# Clinical outcomes and cognitive impairments between progressive supranuclear palsy and multiple system atrophy

**DOI:** 10.1002/brb3.2827

**Published:** 2022-11-21

**Authors:** Peifei Jia, Jinhong Zhang, Jiuyan Han, Yong Ji

**Affiliations:** ^1^ Clinical College of Neurology, Neurosurgery and Neurorehabilitation Tianjin Medical University Tianjin China; ^2^ Department of Neurology The Second Affiliated Hospital of Baotou Medical College Baotou China; ^3^ Department of Neurology Cangzhou People's Hospital Cangzhou Hebei China; ^4^ Department of Neurology, Beijing Tiantan Hospital Capital Medical University Beijing China; ^5^ Department of Neurology Tianjin Huanhu Hospital Tianjin China; ^6^ Department of Neurology, Beijing Tiantan Hospital, Capital Medical University China National Clinical Research Center for Neurological Diseases Beijing China

**Keywords:** cognitive impairments, multiple system atrophy, progressive supranuclear palsy, the mini‐mental state examination, the Montreal Cognitive Assessment

## Abstract

**Background:**

Both progressive supranuclear palsy (PSP) and multiple system atrophy (MSA) belong to atypical parkinsonian syndromes. It is important to differentiate these diseases accurately. We compared clinical outcomes and cognitive impairments between PSP and MSA.

**Methods:**

Eighty‐five MSA parkinsonism type (MSA‐P) patients and 76 PSP patients participated in this research. The Montreal Cognitive Assessment (MoCA) and the mini‐mental state examination (MMSE) evaluated cognitive function.

**Results:**

MSA‐P patients had a significantly higher incidence of dyskinesia, fall, urinary symptoms, and constipation, whereas patients with PSP had a higher incidence of tremor and salivation. MSA‐P patients had higher MMSE and MoCA scores than PSP patients. The MMSE score showed a diagnostic cut‐off score of 24.5 in PSP versus MSA‐P. The MoCA score showed a diagnostic cut‐off score of 20.5 in PSP versus MSA‐P.

**Conclusion:**

In conclusion, patients with PSP had differences in the clinical outcomes and cognitive impairments compared with MSA‐P patients. PSP patients had more severe cognitive deficits. The score of MMSE and MoCA could be used in distinguishing MSA‐P from PSP.

## Graphical abstract

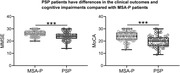

## 1 INTRODUCTION

Multiple system atrophy (MSA) is a neurodegenerative disorder (Fanciulli et al., 2019). Its clinical characteristics include ataxia, parkinsonism, and autonomic failure (Palma et al., 2018). The currently accepted neuropathological hallmark is the misfolded α‐synuclein inclusions in the glial cytoplasm (Wenning & Krismer, 2013). Parkinsonism (MSA‐P) type and cerebellar ataxia (MSA‐C) type are distinguished based on the morphologic phenotypes (Valera & Masliah, 2018). MSA has a low incidence, with an annual incidence of about 3/100,000 in people over 50 (Schrag et al., 1999). The mean onset age of MSA is about 60 years, with no gender differences (Golbe et al., 1988). The prognosis is worse in female patients, older patients, or patients who develop autonomic dysfunction early (O'Sullivan et al., 2008).

One of the other neurodegenerative diseases is progressive supranuclear palsy (PSP) (Armstrong, 2018). The clinical outcomes include Richardson's syndrome, corticobasal syndrome, frontotemporal dementia, parkinsonism, akinesia, and language impairment (Boxer et al., 2017). The incidence of Richardson's syndrome–PSP is about 6/100,000, and the mean onset age is about 60 years (Schrag et al., 1999).

Most neurodegenerative diseases start from the presymptomatic phase, in which neuropathology accumulates but does not yet exceed the threshold required to produce clinical outcomes (Dugger & Dickson, 2017). Both MSA and PSP belong to atypical parkinsonian syndromes, which involve multisystem degeneration and have atypical parkinsonian syndrome features, including dysautonomia, ataxia, ocular dysmotility, frequent falls, and early dementia (Deutschlander et al., 2018). Thus, it is important to differentiate and diagnose these diseases accurately.

This research explored the differences in the clinical outcomes and cognitive impairment between MSA and PSP.

## 2 METHODS

### 2.1 Participants

In this research, participants with PSP or MSA‐P were collected in Beijing Tian Tan Hospital and the Tianjin Huan Hu Hospital from October 2017 to October 2020. Patients with MSA‐P group met the criteria for clinically probable MSA‐P in the diagnosis based on consensus criteria that have been revised in 2008. The patients with PSP group met “probable PSP” criteria in the International Parkinson disease and Movement Disorder Society PSP diagnostic criteria (2017 revision). Exclusion criteria included the presence of cerebrovascular disease, brain inflammation, or drug‐induced Parkinson's syndrome by CT or MRI, significant cortical atrophy on imaging, secondary Parkinson's syndrome, significant depression or current antidepressant medication, and psychiatric illness and inability to complete cognitive testing. The sample size was determined using PS software. Differences of means between MSA‐P and PSP groups were divided by the standard deviation to determine the standardized effect size. Overall, 5% was used as significance level in the Mann–Whitney test; the minimum required sample size was 72 for each group. A total of 85 MSA‐P patients and 76 PSP patients finally participated in this research. Informed consent was derived from each participant. The study was approved by the National Natural Science Foundation (grant number 82172282), Science and Technology Project of Tianjin Municipal Health and Health Committee (grant number ZC20121 and KJ20048).

### 2.2 Data collection

The mini‐mental state examination (MMSE) containing 20 items is used for cognitive impairment assessment. MMSE includes immediate memory, temporal orientation, place orientation, delayed memory, language, visual‐spatial, attention, and computation. The total score of MMSE is 30, and a score of 27–30 is considered normal. MMSE scores below 27 were taken as indicating cognitive impairment.

The Montreal Cognitive Assessment (MoCA) containing 30 items is also used for cognitive impairment assessment. MoCA includes attention and concentration, memory, executive function, visual diagnostic skills, language, abstract thinking, computation, and orientation with a total score of 30, and a score of 26–30 is considered normal.

Both MoCA and MMSE have been translated into Chinese. If participants had less than 12 years of education, they received an additional one point in the MoCA's assessment.

The researchers who conducted and analyzed the questionnaires were blind to the groups.

### 2.3 Statistical analysis

Data were shown as median (interquartile range) or *n* (percentage, %). Fisher's exact test or Chi‐square test was used for assessing the distribution of observations or phenomena between different groups. *p*‐Values for each group were derived from the Mann–Whitney test. Receiver‐operating characteristic (ROC) curves with area under the curve (AUC) (95% CI) were also computed to evaluate the effect of MMSE or MoCA on distinguishing MSA‐P and PSP.

## 3 RESULTS

### 3.1 Baseline characteristics

No significant difference was found in age, duration, smoking history, drinking history, and coronary heart disease history between these two groups (Table 1). PSP patients had higher male proportion than patients with MSA‐P (*p* = .039), higher hypertension (*p* = .002), older onset age (*p* = .012), diabetes incidence (*p* = .007), and lower surgery percentage (*p* < .001) (Table 1).

**TABLE 1:** Baseline characteristics of the patients with multiple system atrophy (MSA) and progressive supranuclear palsy (PSP)

	Study group		
Characteristics	MSA‐P (*n* = 85)	PSP (*n* = 76)	*p*–Value
Age (years)	65 (54, 74)	67 (55, 76)	.153
Gender			
Male	42 (49.4%)	50 (65.8%)	.039
Female	43 (50.6%)	26 (34.2%)	
Age of onset (years)			
<60	53 (62.4%)	32 (42.1%)	.012
≥60	32 (37.6%)	44 (57.9%)	
Duration of disease (years)			
≤3	56 (65.9%)	39 (51.3%)	.164
3–6	13 (15.3%)	18 (23.7%)	
≥6	16 (18.8%)	19 (25%)	
Smoking history			
Yes	21 (24.7%)	15 (19.7%)	.571
No	64 (75.3%)	61 (80.3%)	
Drinking history			
Yes	46 (54.1%)	47 (61.8%)	.342
No	39 (45.9%)	29 (38.2%)	
Hypertension history			
Yes	26 (30.6%)	42 (55.3%)	.002
No	59 (69.4%)	34 (44.7%)	
Diabetes history			
Yes	15 (17.6%)	28 (36.8%)	.007
No	70 (82.4%)	48 (63.2%)	
Coronary heart disease history			
Yes	9 (10.6%)	11 (14.5%)	.482
No	76 (89.4%)	65 (85.5%)	
Operation history			
Yes	28 (32.9%)	7 (9.2%)	<.001
No	57 (67.1%)	69 (90.8%)	

*Note*: Values were expressed as *n* (percentage, %) or median (interquartile range). *p*‐Values for each group were derived from the Mann–Whitney test. Chi‐square test or Fisher's exact test was used for assessing distribution of observations or phenomena between different groups.

### 3.2 The initial symptoms of participants

Comparisons of initial symptoms between the patients with MSA and PSP were also performed. The proportions of glossolalia and memory decline of the two groups had no significant difference (all *p* > .05) (Table 2). MSA‐P patients had significantly higher dyskinesia incidence (*p* < .001), whereas PSP patients had significantly higher tremor incidence (*p* < .001) (Table 2). The main initial symptoms in PSP patients was tremor and in MSA‐P patients was dyskinesia.

**TABLE 2:** Comparisons of initial symptoms between the patients with multiple system atrophy (MSA) and progressive supranuclear palsy (PSP)

	Study group		
Characteristics	MSA‐P (*n* = 85)	PSP (*n* = 76)	*p*‐Value
Glossolalia	5 (5.9%)	6 (7.9%)	.757
Tremor	11 (12.9%)	41 (53.9%)	<.001
Dyskinesia	66 (77.6%)	18 (23.7%)	<.001
Memory decline	2 (2.4%)	3 (3.9%)	.668

*Note*: Values were expressed as *n* (percentage, %). Fisher's exact test was used for assessing distribution of phenomena between different groups.

### 3.3 Non‐motor symptoms of the participants

Comparisons of non‐motor symptoms between the patients with MSA and PSP were also performed. Dysphagia, drinking water cough, hyposmia, and dyssomnia had no significant differences (all *p* > .05) (Table 3). MSA‐P patients had significantly higher fall (*p* < .001), urinary symptoms (*p* = .004), and constipation incidence (*p* = .007), whereas PSP patients had the higher incidence of salivation (*p* = .034) (Table 3).

**TABLE 3:** Comparisons of non‐motor symptoms between the patients with multiple system atrophy (MSA) and progressive supranuclear palsy (PSP)

	Study group		
Characteristics	MSA‐P (*n* = 85)	PSP (*n* = 76)	*p*‐Value
Urinary symptoms	57 (67.1%)	33 (43.4%)	.004
Constipation	49 (57.6%)	27 (35.5%)	.007
Salivation	9 (10.6%)	18 (23.7%)	.034
Dysphagia	14 (16.5%)	16 (21.1%)	.544
Drinking water cough	29 (34.1%)	30 (39.5%)	.515
Fall	54 (63.5%)	24 (31.6%)	<.001
Hyposmia	6 (7.1%)	7 (9.2%)	.774
Dyssomnia	39 (45.9%)	25 (32.9%)	.108

*Note*: Values were expressed as *n* (percentage, %). Fisher's exact test was used for assessing distribution of phenomena between different groups.

### 3.4 Cognitive impairments of the participants

**FIGURE 1:**
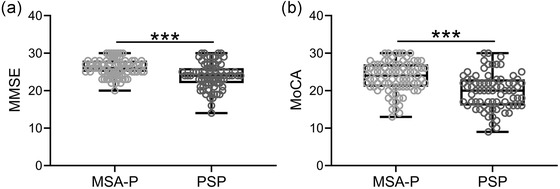
Comparisons of mini‐mental state examination (MMSE, a) and Montreal Cognitive Assessment (MoCA, b) between the patients with multiple system atrophy (MSA) and progressive supranuclear palsy (PSP). Box plot is used to present the data. ***p < .001. Mann–Whitney test

In this research, the cognitive impairments were evaluated by MMSE and MoCA. As shown in Figure 1a, Figure 1b, MSA‐P patients had significantly higher MMSE and MoCA scores than patients with PSP. Thus, cognitive impairment was more severe in PSP patients.

### 3.5 ROC analysis of MMSE and MoCA scores

**FIGURE 2:**
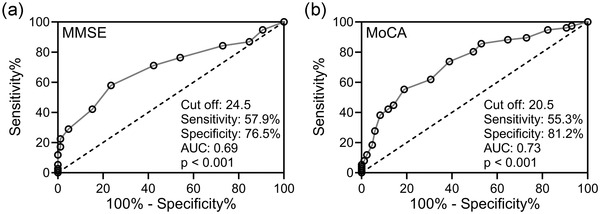
ROC analysis of mini‐mental state examination (MMSE, a) and Montreal Cognitive Assessment (MoCA, b) to differentiate patients with multiple system atrophy (MSA) and progressive supranuclear palsy (PSP)

An ROC analysis was employed to analyze the effects of MMSE and MoCA in the differentiation of patients with MSA and PSP. In PSP versus MSA‐P, diagnostic cut‐off score of MMSE was 24.5 (AUC 0.69, sensitivity 57.9%, specificity 76.5%) (Figure 2). The diagnostic cut‐off score of MoCA was 20.5 (AUC 0.73, sensitivity 55.3%, specificity 81.2%) (Figure 2). Thus, both MMSE and MoCA were effective in the differentiation of patients with MSA and PSP.

### 3.6 Cognitive states of the participants

Table 4 shows the difference in the presence of cognitive impairment between the MSA‐P and PSP groups comparing the MMSE and MoCA. A score of less than 27 and a MoCA of less than 26 were considered the basis for determining cognitive impairment. As shown in Table 4, in the comparisons of MMSE and MoCA scores, PSP patients had a significantly higher proportion of cognitive impairments than MSA‐P patients.

**TABLE 4:** Comparisons of cognitive states between the patients with multiple system atrophy (MSA) and progressive supranuclear palsy (PSP)

		Study group		
Characteristics		MSA‐P (*n* = 85)	PSP (*n* = 76)	*p*‐Value
MMSE	Normal	39 (45.9%)	18 (23.7%)	.005
	Impairment	46 (54.1%)	58 (76.3%)	
MoCA	Normal	31 (36.5%)	9 (11.8%)	<.001
	Impairment	54 (63.5%)	67 (88.2%)	

*Note*: Values were expressed as *n* (percentage, %). Fisher's exact test was used for assessing the distribution of phenomena between different groups.

Abbreviations: MoCA, Montreal Cognitive Assessment; MMSE, mini‐mental state examination.

## 4 DISCUSSION

Both PSP and MSA belong to parkinsonian syndromes (Srivanitchapoom et al., 2018). Because there are no genetic, biochemical, and imaging tests to diagnose or distinguish different diseases in parkinsonian syndromes definitively, the diagnosis of these diseases is entirely clinical (Berardelli et al., 2013). The precise diagnosis is based on the complete medical history, including important clinical signs and the timeline of symptoms (Hughes et al., 2002). According to the complex outcomes, the diagnosis of MSA and PSP is influenced by emerging clinical signs and the clinical experience of specialists (Williams & Litvan, 2013).

The differential diagnosis between different kinds of parkinsonism is critical for therapeutic options. For PSP and MSA, definitive diagnosis contributes to disease course, prognosis, expected clinical progression, and therapeutic modalities. Thus, the differences in the clinical outcomes are essential for the precise diagnosis. In this research, we compared the clinical outcomes between PSA and MSA‐P.

The MSA‐predominant parkinsonism (MSA‐P) is a subtype of MSA (Gilman et al., 2008). Both vegetative symptoms and motor impairment are the main MSA symptoms (Jecmenica‐Lukic et al., 2012) (Erkkinen et al., 2018). Some other symptoms also occur in the patients with MSA‐P, such as dysphagia, inspirational stridor, dysarthria, posture abnormalities, focal dystonias, and sleep behavior disorder (Terao et al., 2016). Structural MRI shows MSA patients’ pons, putamen, cerebellum, and middle cerebellar peduncles atrophy (Brooks et al., 2009).

Patients with PSP have diverse clinical manifestations. Richardson's syndrome is the most common symptom of PSP, which includes levodopa‐resistant akinetic‐rigid symptoms and vertical gaze paresis (Lopez et al., 2016). The other typical symptoms of PSP include frontal lobe syndrome, swallowing impairment, and spastic speech (Armstrong, 2018). The results of structural MRI prove the atrophy of the thalamus, caudate, cerebellum, pons, frontal cortex, dorsal midbrain, and relative subcortical white matter (Graber & Staudinger, 2009). Cortical involvement correlates with cognitive impairment in PSP patients (Ballard et al., 2018).

In this research, we analyzed the baseline characteristics of the participants. By comparison, we found that the proportion of males was higher in PSP patients, and onset age and the proportion of patients with hypertension history or diabetes history were also higher in PSP patients. However, the probability of having a history of surgery was significantly higher in MSA‐P patients.

We also analyzed the initial symptoms of PSP and MSA‐P. These initial symptoms included glossolalia, tremor, dyskinesia, and memory decline. Based on the results, tremor was significantly more frequent in PSP patients, whereas MSA‐P patients had a higher rate of dyskinesia, especially gait disorder and limb inflexibility.

We also compared the non‐motor symptoms. These non‐motor symptoms included urinary symptoms, constipation, salivation, dysphagia, drinking water, cough, fall, hyposmia, and dyssomnia. By comparing the data, we found that MSA‐P patients were more likely to have urinary symptoms, constipation, and falls, whereas PSP patients were more likely to have salivation.

In patients with MSA, cognitive deficits have also been reported (Park et al., 2020). But cognitive impairment is often overlooked because of motor impairment. However, cognitive impairment is identified in nearly 75% of MSA cases (Vecchio et al., 2018).

Most patients with PSP also exhibit cognitive impairment, executive dysfunction, behavioral abnormalities, inefficient memory recall, and personality changes (Litvan et al., 1996). In PSP cases confirmed pathologically, the proportion of patients with cognitive symptoms is 8% at onset and increases to 60% after 3 years (Litvan et al., 1996). Another research has demonstrated that nearly 29% of the PSP cases had cognitive decline within 2 years of disease onset (Williams et al., 2005).

In this research, we also explored the cognitive impairment in both PSP patients and MSA‐P patients. MMSE and MoCA were employed to evaluate the cognitive function. The lower MMSE and MoCA scores in PSP patients indicated that PSP patients had more severe cognitive impairment. Based on MMSE and MoCA scores, the proportion of patients having cognitive impairment was significantly higher in PSP patients than in MSA‐P patients. Thus, we analyzed whether the score of MMSE or MoCA can distinguish PSP and MSA‐P through ROC curves. The results of ROC curves indicated that both MMSE and MoCA scores could differentiate PSP from MSA‐P.

There were some limitations in this research. First, both MSA‐P and PSP were diagnosed based on consensus criteria. We lacked the pathological confirmation of clinical diagnoses. Second, we focused only on parkinsonism (MSA‐P) type, and our findings may not apply to other type of MSA. Third, healthy control subjects should be involved in the research to verify whether cognitive impairments were specific in patients with MSA‐P or PSP. Fourth, because of the ceiling effect of MMSE, early cognitive decline might be ignored in this research.

## 5 CONCLUSION

In conclusion, patients with PSP had differences in the clinical outcomes and cognitive impairments compared with patients with MSA‐P. PSP patients had more severe cognitive deficits than MSA‐P patients. The score of MMSE and MoCA could be used in distinguishing MSA‐P from PSP.

## CONFLICTS OF INTEREST

No conflicts of interest exist.

### PEER REVIEW

The peer review history for this article is available at https://publons.com/publon/10.1002/brb3.2827.

## Data Availability

Data will be made available upon reasonable request to the corresponding author.
